# Antioxidant Activities of Konjac Glucomannan Hydrolysates of Different Molecular Weights at Different Values of pH

**DOI:** 10.3390/foods12183406

**Published:** 2023-09-12

**Authors:** Phattanit Tripetch, Supaporn Lekhavat, Sakamon Devahastin, Naphaporn Chiewchan, Chaleeda Borompichaichartkul

**Affiliations:** 1 Department of Food Technology, Faculty of Science, Chulalongkorn University, Phayathai Road, Patumwan, Bangkok 10330, Thailand; phattanit.t@gmail.com; 2 Thailand Institute of Scientific and Technological Research, 35 Mu 3 Technopolis, Khlong Ha, Khlong Luang, Pathum Thani 12120, Thailand; supaporn_pis@tistr.or.th; 3 Advanced Food Processing Research Laboratory, Department of Food Engineering, Faculty of Engineering, King Mongkut’s University of Technology Thonburi, 126 Pracha U-Tid Road, Tungkru, Bangkok 10140, Thailand; sakamon.dev@kmutt.ac.th (S.D.); naphaporn.rat@kmutt.ac.th (N.C.); 4 The Academy of Science, The Royal Society of Thailand, Dusit, Bangkok 10300, Thailand

**Keywords:** degree of polymerization, enzymatic hydrolysis, Fourier-transform infrared spectroscopy, mannanase, molecular weight distribution, oligosaccharides

## Abstract

Konjac glucomannan (KGM) is a high-molecular-weight polysaccharide that was originally extracted from the corms (underground storage organs) of *Amorphophallus konjac*. KGM and its oligomers have been reported as dietary fibers that exhibit an array of health benefits. The depolymerization of KGM via enzymatic hydrolysis at different conditions gives products of low viscosity and can be used for coating materials in microencapsulation. In the present study, konjac glucomannan hydrolysates (KGMHs) were produced by enzymatic hydrolysis using commercial mannanase at pH 4.5 at 70 °C for 5–120 min, then KGMHs’ molecular weight (*M*_w_), Degree of Polymerization (DP) and their bioactivities were determined. A longer hydrolysis time resulted in KGMH of a lower DP. Oligoglucomannans (*M*_w_ < 10,000) could be obtained after hydrolysis for 20 min. The DP of KGMH rapidly decreased during an early stage of the hydrolysis (first 40 min); DP reached around 7 at the end of the hydrolysis. Antioxidant activities were determined by the DPPH radical scavenging and FRAP assays of KGMHs prepared at pH 4.5 and evaluated at pH 2.0–8.0 depending on pH. KGMH having lower *M*_w_ exhibited higher antioxidant activities. KGMHs having the smallest molecular weight (*M*_w_ = 419) exhibited the highest DPPH radical scavenging activity. *M*_w_ and pH have a greater impact on KGMHs’ bioactivities which can be useful information for KGMHs as functional ingredients.

## 1. Introduction

During the past decades, different forms of oligosaccharides, promoted as functional health ingredients, have been developed and commercialized [1]. While oligosaccharides can be prepared from any sugar monomers, most recent studies have focused on producing and characterizing fructooligosaccharides (FOS) and galactooligosaccharides (GOS) due, for the most part, to their potential as prebiotics. However, oligosaccharides of different molecular weights exhibit different distinct biological functions, such as anti-tumor [2], anti-inflammatory [3], and anti-allergic [4]. Furthermore, Xu et al. (2023) [5] explains that the non-compact structure of oligosaccharides consists of more free hydroxyl (OH) groups and surface area along their structure. These hydroxyl groups can donate electrons, which makes them effective scavengers of free radicals [5]. Free radicals are highly reactive molecules that can cause cellular damage by stealing electrons from other molecules, leading to oxidative stress. Oligosaccharides neutralize free radicals by donating electrons, thus stabilizing the radicals, and preventing them from causing further damage. Therefore, oligosaccharides which have such properties could behave as antiradicals and be used as functional ingredients. Thus, it is of interest to identify a novel raw material that can be conveniently processed into functional oligosaccharides which exhibit biological properties. The understanding of bioactivity from different molecular weights of oligomers, as well as conditions of hydrolysis of high-molecular-weight polymers to oligomers, is crucial to understand the functional properties of oligomers.

Konjac (*Amorphophallus* spp.) is a plant containing large amounts of polysaccharides (90%) that can be used in the production of oligosaccharides. Beside polysaccharides, it also consists of ash (7%), protein (2%) and lipid (0.3%) [6]. The konjac market size was reported to be USD 948.2 Million in 2021 globally, and is expected to reach USD 1.5 Billion by 2028 [7]. Konjac is a rich source of glucomannan, which is a highly viscous soluble dietary fiber. It is a heteropolysaccharide composed of D-glucose and D-mannose units linked by β-1,4 glycosidic bonds with few acetyl groups. Glucomannan sources influence the ratio of glucose and mannose monomers [8]. The molecular weight of native konjac glucomannan (KGM) ranges from 1.0 × 10^5^ to 2.0 × 10^6^ Da and is influenced by the plant species and area of cultivation [9]. KGM has been used as a gelling agent, thickener, film-forming agent and emulsifier in various industries, including food, pharmaceutical and biotechnological [10]. However, the application of high-molecular-weight KGM as a food additive is, in some cases, limited due to its highly viscous nature. Numerous studies have therefore been conducted to reduce the molecular size of KGM to make it more suitable for some specific utilizations and for use as an oligosaccharide. As people have become more health-conscious, seeking out low-calorie, low-carb, and gluten-free alternatives, Konjac-based products have gained popularity because of the consumer demand for healthy and natural food products.

Several means, including subcritical water treatment as well as acid and enzymatic hydrolyses, have been applied to transform KGM into lower-molecular-weight products [9,11,12]. Enzymatic hydrolysis has nevertheless been most widely used to hydrolyze polysaccharides due to its high specificity, safety, predictability and lack of undesirable by-products [13,14]. The most frequently used enzyme for the depolymerization of KGM is mannanase [15,16]. β-1,4-mannanase is an endohydrolase that can randomly cleave within the 1,4-β-D mannan main chain of glucomannan [17]. Properties of the resulting hydrolysates depend on the hydrolysis conditions, including pH, temperature and time [16,18]. Liu et al. (2015) [19], for example, prepared konjac oligoglucomannan using β-mannanase (at enzyme concentration of 150 U/g) at pH 6.0 and 50 °C for 2 h. The average degree of polymerization (DP) of the resulting oligoglucomannan was 5.2. The hydrolysate having a lower viscosity (31.9 mPa·s) was obtained. The product possessed significant antioxidant activity against the hydroxyl radical (•OH) and 1,1-diphenyl-2-picrylhrazyl radical (•DPPH). Wattananprasert et al. (2016) [20] later reported that oligosaccharides with a DP of 4–7 with a viscosity of lower than 100 mPa·s could be obtained after the hydrolysis of KGM powder using mannanase (125 units per g of KGM) at pH 7.1 and 40 °C for 4 h. Very few studies, if any, are available on the antioxidant activities of KGM oligosaccharides of different molecular weights. Furthermore, since hydrolysis conditions also have a significant impact on DP, the physicochemical and functional properties of hydrolysates obtained at different conditions (hence having different molecular weights) are expected to be different. This latter piece of information is also lacking.

Based on the aforementioned arguments, the first aim of the present study was to determine the values of molecular weight (*M*_w_) and DP of konjac glucomannan hydrolysates (KGMHs) prepared via enzymatic hydrolysis using commercial mannanase. The antioxidant activities of KGMHs with varying molecular weights were also evaluated at different pH values to determine their potential use as a functional ingredient in many areas of applications, including food, feed and cosmetic.

## 2. Materials and Methods

### 2.1. Materials and Chemicals

Konjac glucomannan (KGM, 95% purity) was purchased from Yunnan Genyub Konjac Resource Crop (Kunming, China). Food-grade mannanase with an activity of 10,000 U/g was produced by *Bacillus* sp. and was obtained from Amano Enzyme (Nagoya, Japan). Mannose (DP1: *M*_w_ = 180.16), mannobiose (DP2, *M*_w_ = 342.30), mannotriose (DP3, *M*_w_ = 504.40), mannotetraose (DP4, *M*_w_ = 666.60), mannopentaose (DP5, *M*_w_ = 828.70) and mannohexaose (DP6, *M*_w_ = 990.90) were purchased from Megazyme (Bray, Ireland). DPPH (2,2-diphenyl-1-picryl-hydrazyl-hydrate), 6-hydroxy-2,5,7,8-tetramethylchroman-2-carboxylic acid (Trolox) and 2,4,6-tripyridyl-s-triazine (TPTZ) were purchased from Sigma-Aldrich (Singapore).

### 2.2. Preparation of KGMHs of Different Molecular Weights

Mannanase with an activity of 750 U/g (0.075%) was dissolved in 0.1 M sodium acetate buffer (pH 4.5 and 5); the temperature of the solution was controlled at 70 °C by using a water bath. KGM powder was mixed into the mannanase solution to reach a KGM concentration of 10% (*w*/*w*). The contents were maintained at 70 °C under constant stirring at 1000 rpm for either 5, 10, 15, 20, 40, 60 or 120 min. The enzymatic reaction was stopped by boiling each hydrolyzed sample solution for 10 min. The extent of glucomannan hydrolysis was monitored through the determination of the total sugar content (TS) via the sulfuric acid–phenol method, with glucose as the standard [21]. Reducing sugars content (RS) was determined by DNS colorimetry [22]. DP was eventually calculated as DP = TS/RS [19,23].

### 2.3. Determination of Molecular Weight Distribution of KGMHs

The molecular weight distribution of each KGMH was determined using a high-performance liquid chromatograph (Agilent 1100 Series, Agilent Technologies, Santa Clara, CA, USA) equipped with a PL (Polystyrene-Divinylbenzene) aquagel-OH 30 column, 8 μm, 300 × 7.5 mm (Agilent Technologies) and a refractive index detector. The temperature was maintained at 25 °C. Deionized water was used as the mobile phase with no gradient. The injection volume was 50 μL and the flow rate was set as 1 mL/min. Mannose, mannobiose, mannotriose, mannotetraose, mannopentaose and mannohexaose were used as the sugar and oligosaccharide standards to identify the molecular weight of each KGMH. The polydispersity index (PDI) was calculated to determine the distribution width of molecular weights within each KGMH sample, which was calculated as below [24].

PDI = weight-average molecular weight/number-average molecular weight(1)

### 2.4. Fourier-Transform Infrared Spectroscopy

Fourier-transform infrared (FTIR) spectra of KGM and KGMHs were obtained using an FTIR spectrometer (Perkin-Elmer, Spectrum One, Waltham, MA, USA). The KGM solution and KGM hydrolysate solution were analyzed using the ATR–FTIR technique. The solution was dropped on the ATR (Attenuated Total Reflectance) and set to 64 scans, at a resolution of 4 cm^−1^. The spectra were obtained within the wavenumber range of 400–4000 cm^−1^.

### 2.5. Determination of Antioxidant Activities

#### 2.5.1. DPPH Assay

The DPPH radical scavenging activities of KGMHs of different molecular weights (*M*_w_ = 9212, 4342, 629 and 419) at pH 2.0–8.0 were determined using the modified methods of [25] Brand-Williams et al. (1995). DPPH solution was prepared by dissolving 2.5 mg DPPH in 100 mL of ethanol (0.025 g/L). The mixture consisted of DPPH solution with either sample or Trolox solution, and was incubated in the dark at room temperature for 30 min. Absorbance was measured using a UV–vis spectrophotometer (Lambda 25, PerkinElmer, Boston, MA, USA) at 515 nm. A standard curve was prepared using stock solutions of Trolox at the concentrations of 0–140 µM (*y* = 0.1949*x*, R^2^ = 0.99). The results are expressed in mM Trolox/g dried sample.

#### 2.5.2. FRAP Assay

KGMHs of different molecular weights (*M*_w_ = 9212, 4342, 629, and 419) at pH 2.0–8.0 were assayed using the modified methods of [26] Benzie and Strain (1996). The Trolox standard curve was used to determine the FRAP antioxidant activity. In total, 0.0062 g of Trolox was dissolved in 50 mL of 100% methanol to yield 500 μM Trolox solution. The solution was then diluted to 100 μM by adding 10 mL of 500 μM Trolox solution into a volumetric flask and filling it to 50 mL with distilled water. The dilution was repeated using appropriate amounts of distilled water to yield the final concentrations of 20–140 µM (*y* = 0.00305*x*, R^2^ = 0.99).

Prior to adding the FRAP reagent into each KGMH solution, the solution was warmed to 37 °C in a water bath for 10 min. Antioxidant activity was determined by mixing 7.5 mL of the FRAP solution with 0.5 mL of either Trolox solution or sample or distilled water in a test tube using a vortex mixer. After 30 min in the dark at room temperature, the absorbance of the mixture was measured at 593 nm. The value is expressed as mM Trolox/g dried sample [26].

### 2.6. Statistical Analysis

Completely randomized design was used to schedule the experiments with three replications. The results are reported as mean values with standard deviations. Two-way analysis of variance (ANOVA) was performed using SPSS version 22 (IBM SPSS, Chicago, IL, USA) to determine the effect of pH, time, and their interactions to total sugars and reducing sugars contents. Tukey’s test was used to determine the statistical differences among the mean values at a confidence level of 95%.

## 3. Results

### 3.1. Effect of Enzymatic Hydrolysis on TS, RS and DP

Table 1 lists the changes in the contents of total and reducing sugars as well as the degree of polymerization of KGMHs. Note that the ratio of total sugars to reducing sugars was used to calculate DP, and provided the average number of sugar units in a polysaccharide chain. A two-way ANOVA analysis was conducted to assess the impact of different parameters (pH and time) and their interaction.

The analysis found that time had a significant effect on both TS and RS (*p* < 0.05). However, pH only had a significant effect on RS, but not on TS (*p* > 0.05). Similarly, for the interaction, it was observed that there was a significant interaction for RS only. This suggests that the changes in RS levels over time were different depending on pH. An increase in TS and RS resulted in a decrease in DP at an extended hydrolysis time. RS content was in the range of 2.53 ± 0.02 to 8.18 ± 0.01 mg/mL, which corresponded to a decrease in DP from 20 to 7 at pH 4.5. A longer hydrolysis time yielded a higher content of RS and lower DP of KGMH.

### 3.2. Molecular Weight Distribution of KGMHs

Table 2 shows *M*_w_ of KGMHs prepared at different hydrolysis conditions. Hydrolysis time had a significant effect on the depolymerization of KGM. A decrease in the *M*_w_ of KGMH I was rapid during 0–60 min. In the case of KGMH II, the *M*_w_ of the sample declined at a higher rate; a signification reduction was observed within 20 min of the hydrolysis. A similar *M*_w_ of lower than 10^3^ Da to that in the case of KGMH I was noted when the hydrolysis time was beyond 20 min.

### 3.3. FTIR Spectra of KGM and KGMHs

**Figure 1:**
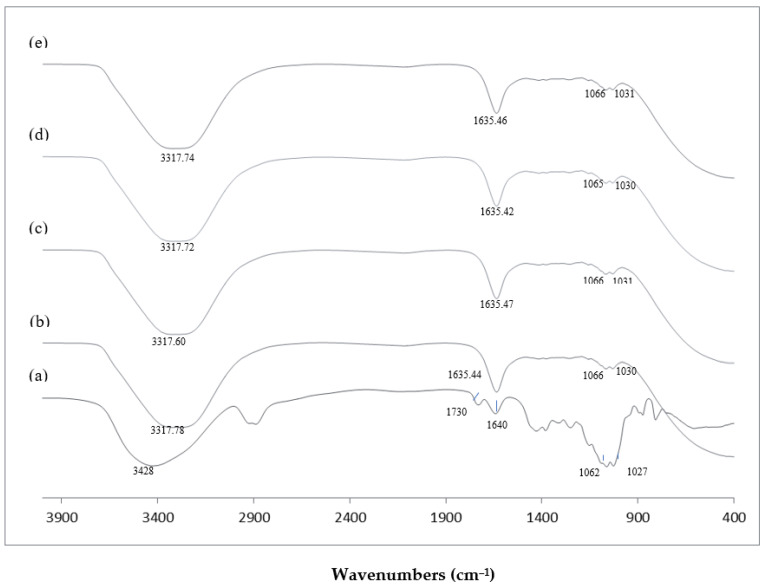
FTIR spectra of KGM and KGMHs prepared at pH 4.5 and 70 °C. (**a**) Native KGM; (**b**) KGMH prepared at hydrolysis time of 20 min, *M*_w_ = 9212; (**c**) KGMH prepared at hydrolysis time of 40 min, *M*_w_ = 4342; (**d**) KGMH prepared at hydrolysis time of 60 min, *M*_w_ = 629; (**e**) KGMH prepared at hydrolysis time of 120 min, *M*_w_ = 419.

FTIR spectroscopy was conducted to observe the modification in the molecular structure of the samples. Figure 1 shows the FTIR spectra of KGM and KGMH I (*M*_w_ of 9212–420 Da).

### 3.4. Antioxidant Activities of KGMHs

**Figure 2:**
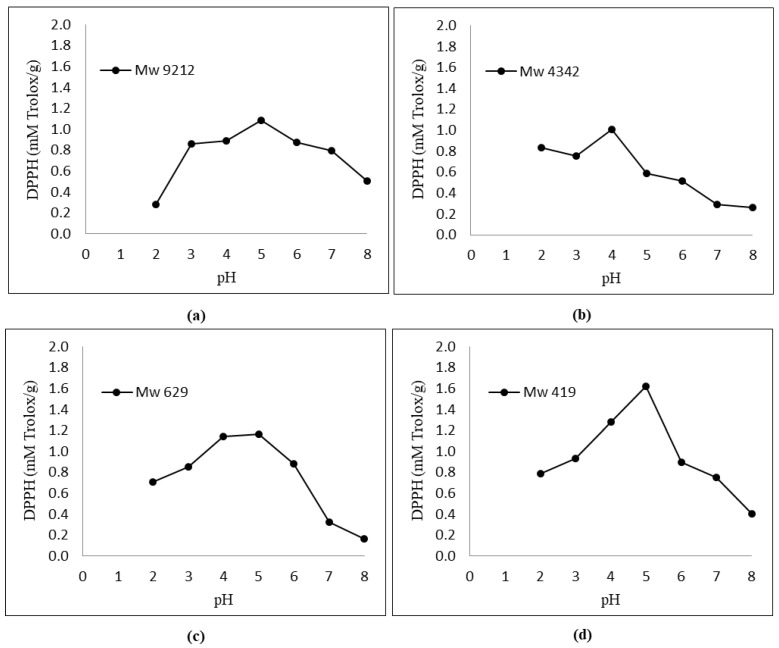
Effect of pH (2.0–8.0) on DPPH radical scavenging activity of KGMHs of different molecular weights. (**a**) *M*_w_ = 9212, (**b**) *M*_w_ = 4342, (**c**) *M*_w_ = 629 and (**d**) *M*_w_ = 419.

Figure 2 shows that the antioxidant activity of KGMHs depended on the pH. An increase in the antioxidant activity was observed when the pH increased from 2 to 5. All the samples exhibited the highest antioxidant activity at pH 5.

**Figure 3:**
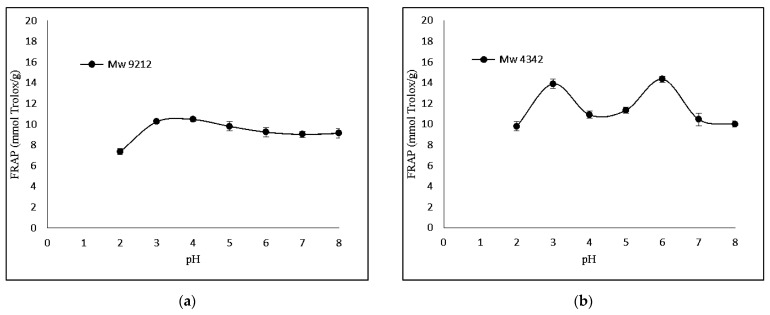

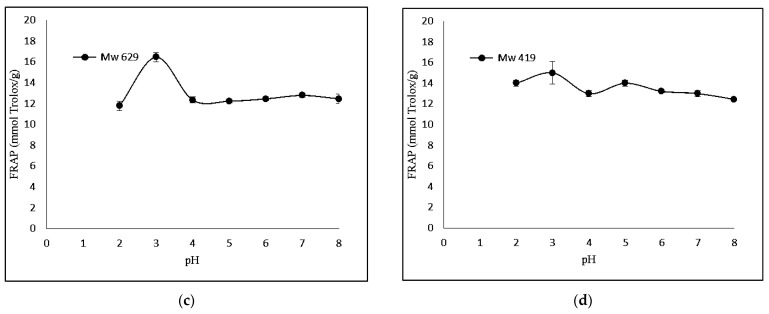
Effect of pH (2.0–8.0) on ferric-reducing antioxidant power of KGMHs of different molecular weights. (**a**) *M*_w_ = 9212, (**b**) *M*_w_ = 4342, (**c**) *M*_w_ = 629 ad (**d**) *M*_w_ = 419.

The FRAP-based antioxidant activity was also determined. Figure 3 shows that KGMH I of smaller molecular weights (*M*_w_ = 629 and 419) exhibited higher levels of ferricreducing capabilities.

## 4. Discussion

### 4.1. Effect of Enzymatic Hydrolysis on TS, RS and DP

KGMH was produced by hydrolyzing KGM with mannanase. The hydrolysis took place via the breaking down of β-1,4-D-mannose and D-glucose in the backbone of KGM. Table 1 lists the changes in the contents of total and reducing sugars as well as the degree of polymerization of KGMHs at different hydrolysis times. An increase in TS and RS resulted in a decrease in DP at an extended hydrolysis time. Mannanase cleaved β-1,4-linked glycosidic bonds, which resulted in the production of reducing sugars, i.e., glucose, mannose and aldose [15]. RS content was in the range of 2.53 ± 0.02 to 8.18 ± 0.01 mg/mL, which corresponded to a decrease in DP from 20 to 7 at pH 4.5. Oligoglucomannans could be obtained at a hydrolysis time of 60–120 min. Enzymatic hydrolysis at pH 5 gave a similar trend, i.e., a longer hydrolysis time yielded a higher content of RS and a lower DP of KGMH. It is also noted that the DP of KGMHs rapidly decreased at an early stage (first 40 min) and reached a value of around 7–8 at the hydrolysis time of 120 min at both pH 4.5 and 5. An increase in pH did not give a significant difference in the DP of the hydrolysates.

### 4.2. Molecular Weight Distribution of KGMHs

To determine the extent to which KGM was hydrolyzed, mono-, di-, tri- and oligosaccharides generated in the hydrolysates were quantified using high-performance liquid chromatography. Note that the molecular weight of KGM is reported to be in the range of 2.476–2.508 × 10^5^ g/mol [27]. The weight-average molecular weight (*M*_w_) and number-average molecular weight (*M*_n_) of KGMHs were measured by quantifying the hydrolysate compositions through HPLC.

Table 2 shows the *M*_w_ of KGMHs prepared at different hydrolysis conditions. The values were estimated using the equivalent RI signals of the standards with known *M*_n_, namely, mannose, mannobiose, mannotriose, mannotetraose, mannopentaose and mannohexaose. Hydrolysis time had a significant effect on the depolymerization of KGM. A decrease in the *M*_w_ of KGMH I was rapid during 0–60 min. The reaction rate slowed down as the time exceeded 60 min. Only slight reduction in *M*_w_ was noted at an extended hydrolysis time (120 min). In the case of KGMH II, the *M*_w_ of the sample declined at a higher rate; a signification reduction was observed within 20 min of the hydrolysis. A similar *M*_w_ of lower than 10^3^ Da to that in the case of KGMH I was noted when the hydrolysis time was beyond 20 min. These results imply that a slight increase in pH exerted a significant effect on the depolymerization rate of KGM.

It was also observed that KGMH I contained polymers with larger PDI values, indicating a larger size distribution (or larger non-uniformity) of the sample. More uniform *M*_w_ was observed at an extended hydrolysis time. KGMH II exhibited lower *M*_w_ and smaller PDI values during the early period of hydrolysis. However, the *M*_w_ of the two samples became closer to each other after being hydrolyzed for 60–120 min. As the polysaccharides were hydrolyzed, the larger and more diverse molecular weight components were likely broken down into smaller and more uniform fragments. This reduction in molecular weight diversity results in a decrease in *M*_w_ and leads to a lower PDI value.

Based on the above-mentioned results, the hydrolysis condition for the preparation of oligoglucomannans was chosen to be pH 4.5. This is because such a condition could provide a series of oligoglucomannans (*M*_w_ < 10,000 Da) having different *M*_w_ after being hydrolyzed for 20–120 min (*M*_w_ in the approximate range of 9212–420 Da).

### 4.3. FTIR Spectra of KGM and KGMHs

FTIR spectroscopy was conducted to observe the modification in the molecular structure of the samples. Figure 1 shows the FTIR spectra of KGM and KGMH I (*M*_w_ of 9212–420 Da). FTIR spectra of KGM exhibited a broad intense peak at 3428 cm^−1^, which is attributed to the stretching of the hydroxyl group (-OH). FTIR spectra of KGMH I also showed a similar broad band of the hydroxyl group with a slight shift of peak (~3317 cm^−1^). The peaks at 2900 cm^−1^ and 1730 cm^−1^ appearing in the spectra of KGM are assigned to the vibration of the CH linkage (C-H) and carbonyl group (C=O), respectively [16]. These two peaks are assigned to the acetyl group [16] in the KGM, which disappeared from the spectra of KGMH I, indicating the transformation of KGM to KGMH likely due to the enzymatic hydrolysis process. Peaks at 1635–1640 cm^−1^ appeared in the spectra of both samples, indicating the presence of adsorbed water molecules [28]. The stretching of intramolecular hydrogen bonds at 1640–1635 cm^−1^ in KGMH was noted to be more extensive than that in KGM. The peaks at 1062–1066 cm^−1^ and at 1027–1031 cm^−1^ represent the C-O linkage of the C-OH group [28].

Parallel to recent studies, the FTIR spectra of previously studied biomaterials exhibited distinct peaks indicative of specific molecular groups and interactions. For instance, [29] Rafe and Razafi (2015) observed basil seed gum, and reported a unique peak at 1603 cm^−1^, a characteristic of carboxylate salt stretching related to its anionic nature. This resonance between the two studies emphasizes the sensitivity of FTIR spectroscopy in elucidating molecular compositions and revealing the presence of specific functional groups within the analyzed biomaterials.

### 4.4. Antioxidant Activities of KGMHs

Figure 2 and Figure 3 show the respective antioxidant activities of KGMH for DPPH and FRAP assays. Antioxidant activities were determined at pH 2.0–8.0 to cover the pH range encountered in food, feed and cosmetic products. The results show that the antioxidant activity of KGMHs depended on pH. An increase in the antioxidant activity was observed when the pH increased from 2 to 5. All the samples exhibited the highest antioxidant activity at pH 5. However, the activity decreased at a pH beyond 5. This is consistent with the findings of previous studies, which have shown that the antioxidant activity of oligosaccharides is pH-dependent [16,30]. The optimal pH for the antioxidant activity of oligosaccharides is typically in the neutral to slightly acidic range. This is because the ionization state of the hydroxyl groups on the oligosaccharides is most favorable in this pH range. At a lower pH, the hydroxyl groups are protonated and less likely to donate electrons to free radicals. At a higher pH, the hydroxyl groups are deprotonated and more likely to donate electrons to free radicals [31].

Interestingly, FRAP showed the highest relative ferric reducing capabilities at pH 3. The difference in antioxidant activity between the two methods can be explained by the different principles of the methods. The DPPH radical scavenging assay measures the ability of an antioxidant to scavenge a stable free radical, DPPH, while the FRAP assay measures the ability of an antioxidant to reduce Fe3+ to Fe2+. A FRAP assay is designed to work optimally at lower pH levels (typically at 3.6). At this pH level, the reduction reaction of the ferric–tripyridyltriazine complex by antioxidants is known to be optimized, which could explain the highest antioxidant activity of samples at pH 3 in a recent study [32].

KGMH I with lower *M*_w_ exhibited higher antioxidant activity for both DPPH and FRAP assays, with the highest activity shown by the smallest molecular weights (*M*_w_ = 419 for DPPH; *M*_w_ = 629 and 419 for FRAP). This is expected as polysaccharides with lower molecular weights typically exhibit higher water solubility and can more easily react with free radicals [33]. The higher antioxidant activity of KGMH I with lower *M*_w_ in the DPPH radical scavenging assay is likely because these oligosaccharides have a higher number of free hydroxyl groups. These free hydroxyl groups can donate electrons to the DPPH radical, scavenging it. The higher antioxidant activity of KGMH I with lower *M*_w_ in the FRAP assay is likely because these oligosaccharides are more easily oxidized by Fe3+ [5].

## 5. Conclusions

In this study, KGMHs with different molecular weights (*M*_w_) were successfully prepared via enzymatic hydrolysis using commercial mannanase. The hydrolysis conditions of pH 4.5, 70 °C and 20–120 min were found to be optimal for producing KGM oligosaccharides (*M*_w_ < 10,000) with DP 7 to 12. The antioxidant activity of KGMHs was found to be dependent on both Mw and pH, with lower molecular weights and lower pH values resulting in higher antioxidant activity. These results suggest that KGMHs have potential as functional ingredients in many areas of application, including food, feed and cosmetic.

## Figures and Tables

**Table 1:** Changes in total sugars and reducing sugars contents as well as degree of polymerization during hydrolysis of KGMH.

Condition (pH)	Time (min)	Total Sugars Content (mg/mL)	Reducing Sugars Content (mg/mL)	Degree of Polymerization
4.5	5	50.94 ± 0.16 ^c^	2.53 ± 0.02 ^l^	20
	10	51.34 ± 0.35 ^c^	3.48 ± 0.01 ^j^	15
	15	54.11 ± 0.19 ^b^	3.14 ± 0.01 ^k^	17
	20	56.28 ± 0.28 ^a^	4.51 ± 0.05 ^g^	12
	40	56.21 ± 0.70 ^a^	5.25 ± 0.01 ^f^	11
	60	56.59 ± 0.11 ^a^	6.09 ± 0.01 ^d^	9
	120	56.36 ± 0.33 ^a^	8.18 ± 0.01 ^a^	7
5.0	5	51.45 ± 0.05 ^c^	3.45 ± 0.01 ^j^	15
	10	51.01 ± 0.22 ^c^	3.11 ± 0.02 ^k^	16
	15	53.46 ± 0.20 ^b^	3.98 ± 0.02 ^h^	13
	20	56.15 ± 0.11 ^a^	3.69 ± 0.02 ^i^	15
	40	56.42 ± 0.12 ^a^	5.94 ± 0.03 ^e^	9
	60	56.35 ± 0.11 ^a^	6.72 ± 0.01 ^c^	8
	120	56.31 ± 0.11 ^a^	7.25 ± 0.04 ^b^	8

Mean values within a column followed by the same superscript letters are not significantly different (*p* > 0.05).

**Table 2:** Relative molecular weights of KGMHs at different hydrolysis time.

Sample	pH	Time (min)	*M*_w_ ^1^	*M*_n_ ^2^	PDI ^3^
KGMH I	4.5	5	51,161.95 ± 858.90	10,602.11 ± 173.20	4.83 ± 0.16
		10	41,899.02 ± 757.56	9365.31 ± 463.82	4.47 ± 0.08
		15	39,482.22 ± 398.42	8501.55 ± 206.61	4.65 ± 0.07
		20	9212.27 ± 439.31	3724.17 ± 121.41	2.48 ± 0.20
		40	4342.93 ± 139.43	2725.30 ± 37.76	1.59 ± 0.03
		60	629.49 ± 12.66	1504.27 ± 133.38	0.42 ± 0.03
		120	419.71 ± 34.57	500.67 ± 3.30	0.84 ± 0.06
KGMH II	pH 5	5	12,788.34 ± 464.86	7173.41 ± 134.59	1.78 ± 0.03
		10	7718.89 ± 333.77	5179.61 ± 114.03	1.49 ± 0.10
		15	3749.82 ± 404.42	1542.40 ± 16.98	2.43 ± 0.29
		20	940.29 ± 42.43	1486.25 ± 13.94	0.63 ± 0.02
		40	582.18 ± 15.25	726.84 ± 5.63	0.80 ± 0.01
		60	523.72 ± 17.67	429.21 ± 12.54	1.22 ± 0.01
		120	478.34 ± 5.80	398.74 ± 12.71	1.20 ± 0.05

*M*_W_ is reported in relative to mannose, mannobiose (DP2), mannotriose (DP3), mannotetraose (DP4), mannopentaose (DP5) and mannohexaose (DP6) standards. ^1^ *M*_w_ = weight-average molecular weight. ^2^ *M*_n_ = number-average molecular weight. ^3^ PDI = polydispersity index (*M*_w_/*M*_n_).

## Data Availability

The data are available from the corresponding author.
